# Patterns of computed tomography utilisation in injury management: latent classes approach using linked administrative data in Western Australia

**DOI:** 10.1007/s00068-023-02303-y

**Published:** 2023-06-15

**Authors:** Ninh T. Ha, Mark Harris, Max Bulsara, Jenny Doust, Sviatlana Kamarova, Donald McRobbie, Peter O’Leary, Paul M. Parizel, John Slavotinek, Cameron Wright, David Youens, Rachael Moorin

**Affiliations:** 1 Health Economics and Data Analytics, Curtin School of Population Health, Faculty of Health Sciences, Curtin University, GPO Box U1987, Perth, WA 6845 Australia; 2 School of Accounting, Economics and Finance, Faculty of Business and Law, Curtin University, Perth, Western Australia Australia; 3 Institute for Health Research, University of Notre Dame, Fremantle, WA Australia; 4 Centre for Health Services Research, School of Population and Global Health, The University of Western Australia, Crawley, Australia; 5 Australian Women and Girls’ Health Research Centre, School of Public Health, University of Queensland, Brisbane, Australia; 6 School of Health Sciences, University of Sydney, Camperdown, New South Wales Australia; 7 Nepean Blue Mountains Local Health District, Kingswood, New South Wales Australia; 8 School of Physical Sciences, University of Adelaide, Adelaide, Australia; 9 Obstetrics and Gynaecology Medical School, Faculty of Health and Medical Sciences, The University of Western Australia, Perth, WA Australia; 10 PathWest Laboratory Medicine, QE2 Medical Centre, Nedlands, WA Australia; 11 Medical School, University of Western Australia, Perth, WA Australia; 12 Department of Radiology, Royal Perth Hospital, Victoria Square, Perth, WA 6000 Australia; 13 SA Medical Imaging, SA Health and College of Medicine and Public Health, Flinders University, Adelaide, South Australia Australia; 14 Fiona Stanley Hospital, 11 Robin Warren Dr, Murdoch, WA Australia; 15 Division of Internal Medicine, Medical School, Faculty of Health and Medical Sciences, University of Western, Perth, Australia; 16 School of Medicine, College of Health and Medicine, University of Tasmania, Hobart, TAS Australia

**Keywords:** Computed tomography, Trajectory of CT scanning, injury, high use CT scanning

## Abstract

**Purpose:**

Whilst computed tomography (CT) imaging has been a vital component of injury management, its increasing use has raised concern regarding ionising radiation exposure. This study aims to identify latent classes (underlying patterns) of CT use over a 3-year period following the incidence of injury and factors predicting the observed patterns.

**Method:**

A retrospective observational cohort study was conducted in 21,544 individuals aged 18 + years presenting to emergency departments (ED) of four tertiary public hospitals with new injury in Western Australia. Mixture modelling approach was used to identify latent classes of CT use over a 3-year period post injury.

**Results:**

Amongst injured people with at least one CT scan, three latent classes of CT use were identified including a: temporarily high CT use (46.4%); consistently high CT use (2.6%); and low CT use class (51.1%). Being 65 + years or older, having 3 + comorbidities, history with 3 + hospitalisations and history of CT use before injury were associated with consistently high use of CT. Injury to the head, neck, thorax or abdomen, being admitted to hospital after the injury and arriving to ED by ambulance were predictors for the temporarily high use class. Living in areas of higher socio-economic disadvantage was a unique factor associated with the low CT use class.

**Conclusions:**

Instead of assuming a single pattern of CT use for all patients with injury, the advanced latent class modelling approach has provided more nuanced understanding of the underlying patterns of CT use that may be useful for developing targeted interventions.

**Supplementary Information:**

The online version contains supplementary material available at 10.1007/s00068-023-02303-y.

## Introduction

Globally, injury is one of the leading causes of mortality in adults [1]. Computed tomography (CT) imaging is a vital component of diagnosis and management for most injuries [2], especially in the emergency department (ED) since CT offers high sensitivity and specificity in the evaluation of trauma patients. A Western Australian study showed that the rate of CT examinations in EDs doubled from 58 to 105 per 1000 ED presentations between 2003 and 2015, with injury being the second most likely reason for CT use [3].

The increasing volume of these examinations has raised concerns for patients [4], and is receiving increased attention from health care providers, regulators and the media [4, 5] due to its association with healthcare expenditure and unnecessary exposure to ionising radiation [6]. Although CT scans comprise a small proportion of all diagnostic radiological procedures, a report from the USA in 2009 found that CT and nuclear medicine accounted for 36% of the total radiation exposure and 75% of the medical radiation exposure of the population [7]. The UK review in 2011 shows that typical radiation effective dose for common CT scans in adults increased between 20% (head) to 400% (high-resolution chest) compared to the dose observed in the review in 2003 [8, 9]. Risks are accentuated in trauma patients who are potentially more radiosensitive due to their relatively younger age compared with patients presenting for medical conditions [10], notwithstanding evidence showing that the average age of trauma patients is increasing [11]. On average, trauma patients received a mean effective dose of 22.7 mSv which is almost ten times the average annual background radiation dose (2.4 mSv) [10]. Therefore, monitoring long-term CT use is important for understanding the appropriateness of current imaging practice. Assessment of change in use of CT scanning incorporating characterisation of patients with different patterns of use could help policy-makers construct a comprehensive strategy to evaluate population risk. The finding may also influence clinicians employing CT scanning to consider the individual risks of excessive ionizing radiation to their patients.

Current studies generally focus on examining the use of CT during a single life event, such as an ED presentation [3, 12–15] or hospital admission [16, 17], over short periods. Patients who had substantial increase in CT use following the life event may not be captured in the short periods of observation. Availability of large linked administrative data at the individual level has provided opportunity to use advanced analytic methods such as trajectory modelling techniques to identify unobserved patterns (latent classes) within the population over time [18, 19]. Patterns are defined directly from the data rather than using arbitrarily pre-specified thresholds [19]. In this study, we used mixture modelling of linked administrative data in Western Australia (WA) to empirically identify discrete underlying patterns of CT use over a 3-year period following an initial injury requiring assessment in an ED and factors predicting these patterns.

## Methods

This was a retrospective observational cohort study reported following the Reporting of studies conducted using Observational Routinely-Collected health data (RECORD) statement [20].

### Data source

The study used de-identified individual level health administrative data linked by the WA Data Linkage System and the Australian Institute for Health and Welfare pertaining to adults aged 18 years and over who had any hospitalisation (except for pregnancy), ED presentation or CT scan in WA between 1 January 2003 and 31 December 2016 [21]. For the cohort, the following data were available: (i) WA Public Hospital ED data collection (EDDC) (2003–2016) which provided details on all ED presentations at all public hospital EDs in WA; (ii) WA hospital morbidity data collection (HMDC) data which contained information related to inpatient care in all hospitals (public and private) in WA between 2003 and 2016; (iii) Information on all CT scans performed in WA was sourced from: [1] Picture Archival communication System (PACS) records from 2003 to 2015 which contained records of CT undertaken in all tertiary public hospitals and the majority of secondary public hospitals in WA; and [2] Medicare Benefits Schedule (MBS) claim items from 2005 to 2015 for CT scans for subsidised under Australia's universal health insurance scheme Medicare and undertaken for private patients in private and public hospitals and community-based radiology practices. (iv) The WA Death Registry of Births, Deaths and Marriages records from 2004 to 2015 containing information on all deaths in including the age, date and cause of death. Further details of each datasets are published elsewhere [22].

### Study population

This study included patients aged 18 years or older who was: (1) presenting to any of the four tertiary (teaching or major) hospital EDs in WA; and (2) with a first-time (incident) injury in 2012, defined as no history of injury recorded in both ED and HMDS data over a 3-year lookback period prior to their injury record in 2012.

The restriction to tertiary hospital EDs was made to ensure a consistent cohort could be captured and that initial CT scanning in the ED could be comprehensively identified. This was necessary because (i) private hospital EDs are not included in the ED data collection and (ii) the PACS data does not capture all secondary (district or regional) hospitals. Injured patients were identified using the International Statistical Classification of Diseases, tenth revision, Australian modification (ICD-10-AM) code S00-T14 recorded in the diagnosis field of the ED data [23].

## Use of CT over three-year following the incident injury

All CT scans from the date of ED presentation for the incident injury to 3 years post injury or dead whichever comes first were included. CT scans recorded on the same day and the same anatomical area were counted as one to avoid over counting. The number of CT scans was summed by quarter over the three-year follow-up period. Amongst individuals who had at least one CT scan over the follow-up period, Box–Cox transformation was applied to produce more normally distributed data for use in modelling latent classes of CT use [24]. Individuals with no CT scans over the whole study period were grouped as “no CT use” group and excluded from trajectory modelling for computational efficiency (*i.e.* to improve convergence and time).

## Potential predictors

The following variables were captured: sex; age at incident injury ED presentation (classified into 18–24 years, 25–64 years, 65–79 years and 80 + years) and Indigenous status (used only for adjustment of confounding in models, not reported due to ethics approval conditions). Socio-economic status, in quintiles, was derived from postcode of residence at time of ED presentation using the socio-economic index for areas (SEIFA) index of relative socio-economic disadvantage [25]. Patient’s residential postcode at incident ED presentation was classified into major cities, inner regional areas, outer regional areas, remote and very remote areas according to the Accessibility and Remoteness Index of Australia (ARIA) [26]. External cause of injury was sourced from ED data and was categorised into four main groups after consultation with local ED physicians. The groups were transport/pedestrian, fall, force (blunt force, cut/pierced or stabbed, shot by weapon and contact with machinery) and others (bite or sting, contact burn, contact with fire or flame, exposure or poisoning by chemicals, electrocution, other cause and unknown) and published elsewhere [22]. Anatomical area of injury was classified as head, neck, thorax, abdomen, extremity and multiple injuries. Patients were classified as either admitted to hospital or discharged following the incident ED presentation since those who died in ED were excluded from the cohort. Severity of injury was evaluated using the ICD-10-AM–based Injury Severity Score (ICISS) which has been evaluated internationally and in Australia [23, 27–29] and classified into mild (survival > 99%), moderate (survival > 94%) and severe/very severe (survival < 94%). Details of how severity scores were derived have been published previously [15]. Date of ED presentation was classified into weekday and weekend/public holiday. Mode of arrival was classified into three groups: private transport, ambulance (including air ambulance) and other. ED presentation time was classified into three-time blocks: day (8:00–15:59), evening (16:00–23:59) and night (0:00–7:59). Length of stay was the number of days between admitted date and discharged date of the hospitalisation associated with the incident ED presentation with those not admitted given the value of 0. History of comorbidity was based on the number of comorbidities captured in HMDC records within 5 years prior to the incident injury, classified as none, 1–2 and 3 + comorbidities using the Multipurpose Australian Comorbidity Scoring system [30] using ICD-AM-10 across all diagnostic fields. History of CT scanning was classified as having at least one CT scan recorded in the year prior to the index injury. Number of ED presentations and hospitalisations related to injury during the follow-up period were also captured to adjust for the potential impact of the new event.

### Statistical analysis

Descriptive statistics were used to describe socio-demographic and clinical characteristics of the study cohort at baseline—the time presenting to tertiary ED for the incident injury. We followed the three-step approach [31] including: (1) determining number of classes; (2) assigning the most likely class membership to the study cohort; and (3) a subsequent separate analysis using a multinomial logistic regression model to identify factors predicting different CT use classes.

For identifying latent classes, we utilised finite mixture models (FMMs), originally from Pearson and Henrici [32]. Generically, the approach involves endogenously, and probabilistically, splitting the sample into a finite number of discrete latent classes. Within each class, observations are relatively homogenous, but potentially heterogeneous across them. The probabilistic splitting of the sample is usually achieved by employing multinomial logit (MNL) techniques. Within each class, the same statistical model applies, but are characterised by differing parameters of that density. Importantly, in this way, the same covariates can have differing effects on the outcome variable within each class. The optimal number of classes is usually determined by information criteria and/or entropy [33]. Posterior probabilities are used to predict class membership of individuals (based on the maximum probability rule).

The defined classes are “labelled” to reflect the typical composition of the class members with respect to the outcome variable ( (i) total number of CT scans within a class to reflect total burden of CT use in each class, (ii) average number of CT scans in an individual within each class to capture burden at individual level, and (iii) time between the first and last CT to indicate level of intensity in exposing to CT scan as suggested by previous publication [34]. These indicators were used to support interpretation of the classes.

In addition to describing the classes by simple descriptive statistics, multinomial logistic regression model, a more nuanced approach, was used to the explain the posterior probabilities—which drive class membership—by a range of predictor variables. No CT use group was acted as the reference (i.e. control group) in the model to estimate the relative risk of being classified into other CT use class across a range of the predictors. The analysis was conducted using the *lcmm* package under R version 4.0 [35] and STATA MP Version 16 [36].

## Results

### Cohort characteristics

A total of 21,544 individuals aged 18 years and older were identified with an incident injury presenting to a tertiary level ED in 2012. The majority of the study cohort were male (58.2%), aged less than 45 years old (60.7%), and lived in a major city (93.5%) (Table 1). People with a socio-economic status of least disadvantaged accounted for 43.1%. Injuries involving extremities accounted for the highest proportion (64.6%), followed by head injuries (18.9%) and abdominal injuries (5.9%). Most individuals presented with mild injury (92.4%) and had no comorbidities (53.8%).

**Table 1:** Cohort characteristics at the baseline 2012

	*N*	%
Sex		
Male	12,531	58.16
Female	9013	41.84
Age group		
18–29 years	7481	34.72
30–44 years	5606	26.02
45–64 years	4908	22.78
65 + years	3549	16.47
SEIFA		
Least disadvantage	9285	43.10
Less disadvantage	3577	16.60
Moderate disadvantage	4901	22.75
High disadvantage	2340	10.86
Highest disadvantage	1276	5.92
Unknown	165	0.77
ARIA		
Major cities	20,150	93.53
Inner regional areas	427	1.98
Outer regional areas	445	2.07
Remote areas	306	1.42
Very remote areas	131	0.61
Unknown	85	0.39
Anatomical areas of injury		
Head	4077	18.92
Neck	699	3.24
Thorax	1037	4.81
Abdomen	1271	5.90
Extremity	13,928	64.65
Multiple injury	532	2.47
Injury with hospital admission		
No	14,870	69.02
Yes	6674	30.98
Group of external causes of injury		
Transport/pedestrian	2,348	10.90
Fall	5556	25.79
Force^$^	7302	33.89
Others/unknown^$$^	6338	29.42
Severity of injury		
Mild > 99%SRR)	20,022	92.94
Moderate 94.1–99% SRR)	846	3.93
Severe/very severe < = 95% SRR)	676	3.14
Presentation day		
Weekday	13,330	61.87
Weekend/PH	8214	38.13
ED shift		
Day 8:00–15:59)	10,232	47.49
Evening 16:00–23:59)	8169	37.92
Night 0:00–7:59)	3143	14.59
Mode of arrival		
Private transport	15,459	71.76
Ambulance	5766	26.76
Others	319	1.48
Median Length of hospital stay (days)	0	0–1
History of comorbidities		
None	11,592	53.81
1–2 comorbidities	4798	22.27
3 + comorbidities	5154	23.92
History of hospital admission (1 year prior)		
No hospitalisation	13,050	60.57
1–2 hospitalisations	6982	32.41
3 + hospitalisation	1512	7.02
History of CT scan (1 year prior)		
None	19,722	91.54
1 + CT scans	1822	8.46
CT scan use status during 3-year follow-up		
No CT use	14,453	67.10
At least one CT scanning	7091	32.90

*SEIFA* Socio-economic Index for areas, *ARIA* Accessibility and Remoteness Index of Australia, *PH* Public holiday, *IQR* the interquartile range

^$^Force includes blunt force, cut/pierced or stabbed, shot by weapon and contact with machinery

^$$^Others/unknown includes bite or sting, contact burn, contact with fire or flame, exposure or poisoning by chemicals, electrocution, other cause and unknown

### Latent classes of CT use over three-year follow-up

**Fig. 1:**
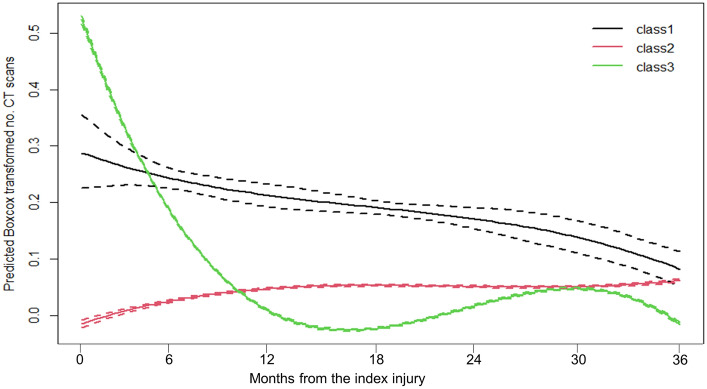
Predicted class trajectory amongst injury patients with at least one CT scan over 3 years

Based on entropy and information criteria, the model with three classes was preferred, with patterns of CT use presented in Fig. 1 (details model output is presented in Appendix 1). The three classes were characterised descriptively according to the pattern of CT use as follows: class 1 –termed “ consistently high use” (2.6% of patients having at least one CT scan over the 3-year follow-up ~ 0.8% of the whole cohort) had a consistently high (but slightly diminishing) use of CT across the study period with no peaks or troughs; class 2—“low use” (51.1% amongst those with at least one CT scan over the 3-year follow-up ~ 16.9% of the cohort) had no CT use at the beginning and a slight increase over the follow-up time; and class 3—“temporarily high use” (46.3% of people with at least one CT scan over the 3-year follow-up ~ 14.6% of the whole cohort) in which the CT use was very high at the start, declining to zero at the end of first year followed by an increase in CT use after 2.5 years. Whilst class 1, “consistently high use”, accounted for nearly 1% of the study population, it accounted for 10% of the total CT use in the cohort (Table 2). Notably the average number of CTs per individual was the highest (7.9 CT scans [95%CI 7.29; 8.45]) of all classes. In contrast, class 2, the “low use” and class 3, the “temporarily high use”, had an average of 1.7 CT scans [95% CI 1.64; 1.73] and 2.2 CT scans [95% CI 2.16; 2.28], respectively (Table 2). As noted in Table 2, 67% of people presenting to a WA tertiary ED with an incident injury had no CT scanning undertaken within three years of the date of presentation.

**Table 2:** Characteristics of CT use classes identified from the finite mixture models amongst people with at least one CT scan over the 3-year follow-up period

CT use classes	N (%)	Total no. of CT (N, %)	Average number of CT in 3 years (Mean (95%CI))/median (IQR)	Days between the first and the last CT
1- Consistently high CT use	181 (2.6)	1425 (9.6)	7.9 (7.29–8.45)/ 7 (5–9)	Mean (SD): 646.1 (269.3) Median (IQR): 659 (397)
2- Low CT use	3621 (51.1)	6111 (41.2)	1.7 (1.64–1.73)/ 1 (1–2)	Mean (SD): 358.7 (275.5) Median (IQR): 315 (455)
3- Temporally high CT use	3289 (46.3)	7297 (49.2)	2.2 (2.16–2.28)/ 2 (1–3)	Mean (SD): 456.2 (366.8) Median (IQR): 430 (720)
	7091 (100)	14,833 (100)	2.1 (2.05–2.14)	

## Factors predicting classes of CT use

Baseline characteristics of the patients in each latent class are presented in Table 3. There were significant differences in age and sex distribution between no CT use and the CT use classes. The majority of the no CT use group were male (60.3%) and aged under 45 years (70.1%). In contrast, class 1—the consistently high use of CT, was characterised by equal distribution between sexes and a high proportion of people aged 65 + years (63.0%). Both class 2 and class 3 had a similar distribution across the age groups, whilst class 3 had a significantly higher proportion of males. In terms of clinical characteristics, individuals in class 1 and class 3 had a significantly higher proportion of head injuries, hospital admissions for the incident injury, injuries due to falls and moderate or severe injuries than those in either class 2 or the no CT use group. Class 1 had the highest proportion of people with 3 + comorbidities (70.0%) compared with classes 2 and 3 (38.6% and 35.1%, respectively).

**Table 3:** Characteristics of patients in No CT use and in each CT use class

Characteristics	No CT use		CT use						*p*-value
			Class 1		Class 2		Class 3		
	*n*	(%)	*n*	%	*n*	%	*n*	%	
Sex									
Male	8713	60.3	90	49.7	1826	50.4	1902	57.8	< 0.001
Female	5740	39.7	91	50.3	1795	49.6	1387	42.2	
Age group									
18–29 years	6052	41.9	6	3.3	705	19.5	718	21.8	< 0.001
30–44 years	4080	28.2	15	8.3	803	22.2	708	21.5	
45–64 years	2978	20.6	46	25.4	1047	28.9	837	25.5	
65 + years	1343	9.3	114	63.0	1066	29.4	1,026	31.2	
SEIFA									
Least disadvantage	6380	44.1	73	40.3	1460	40.3	1372	41.7	< 0.001
Less disadvantage	2418	16.7	28	15.5	613	16.9	518	15.8	
Moderate disadvantage	3265	22.6	44	24.3	896	24.7	696	21.2	
High disadvantage	1470	10.2	21	11.6	406	11.2	443	13.5	
Highest disadvantage	814	5.6	12	6.6	219	6.1	231	7.0	
ARIA*									
Major cities	13590	94.0	169	93.4	3,422	94.5	2969	90.3	< 0.001
Inner regional areas	277	1.9	*		61	1.7	*		
Outer regional areas	282	2.0	*		66	1.8	*		
Remote areas	175	1.2	*		46	1.3	*		
Very remote areas	79	0.6	*		13	0.4	*		
Anatomical areas of injury									
Head/neck	2893	20.0	48	26.5	626	17.3	1209	36.8	
Thorax	613	4.2	15	8.3	202	5.6	207	6.3	
Abdomen	685	4.7	21	11.6	239	6.6	326	9.9	
Extremity	9,973	69.0	88	48.6	2473	68.3	1394	42.4	
Multiple injury	289	2.0	9	5.0	81	2.2	153	4.7	
Admitted to hospital									
No	11264	77.9	72	39.8	2529	69.8	1005	30.6	< 0.001
Yes	3189	22.1	109	60.2	1,092	30.2	2284	69.4	
External cause of injury									
Transport/pedestrian	1329	9.2	23	12.7	311	8.6	685	20.8	< 0.001
Fall	3233	22.4	78	43.1	1073	29.6	1172	35.6	
Force	5639	39.0	22	12.2	1053	29.1	588	17.9	
Others/unknown	4252	29.4	58	32.0	1184	32.7	844	25.7	
Severity of injury									
Mild (> 99%SRR)	13921	96.3	139	76.8	3390	93.6	2572	78.2	< 0.001
Moderate (94.1–99% SRR)	285	2.0	24	13.3	124	3.4	413	12.6	
Severe/very severe (< = 95% SRR)	247	1.7	18	9.9	107	3.0	304	9.2	
Date of presentation									
Weekday	8815	61.0	130	71.8	2343	64.7	2042	62.1	< 0.001
Weekend/PH	5638	39.0	51	28.2	1278	35.3	1247	37.9	
ED shifts									
Day (8:00–15:59)	6791	47.0	91	50.3	1,879	51.9	1,471	44.7	< 0.001
Evening (16:00–23:59)	5533	38.3	75	41.4	1,293	35.7	1,268	38.6	
Night (0:00–7:59)	2129	14.7	15	8.3	449	12.4	550	16.7	
Mode of arrival									
Private transport	11421	79.0	77	42.5	2566	70.9	1395	42.4	< 0.001
Ambulance	3032	21	104	57	1055	29	1894	58	
Median length of stay of an index event (days)	0	0–0	1	0–5	0	0–1	2	0–5	< 0.001
History of comorbidity									
None	8822	61.0	25	13.8	1320	36.5	1425	43.3	< 0.001
1–2 comorbidities	3152	21.8	30	16.6	905	25.0	711	21.6	
3 + comorbidities	2479	17.2	126	69.6	1396	38.5	1153	35.1	
History of hospitalisation									
No hospitalisation	10097	69.9	39	21.6	1,978	54.6	936	28.5	< 0.001
1–2 hospitalisations	3842	26.6	71	39.2	1284	35.5	1785	54.3	
3 + hospitalisation	514	3.6	71	39.2	359	9.9	568	17.3	
History of CT scan									
None	13 735	95.0	105	58.0	3060	84.5	2822	85.8	< 0.001
1 + CT scans	718	5.0	76	42.0	561	15.5	467	14.2	

^*^Low cell count is not allowed to present

*SEIFA* Socio-economic Index for areas, *ARIA* Accessibility and Remoteness Index of Australia, *PH* Public holiday, *IQR* the interquartile range

After adjustment for all observed baseline demographic and clinical characteristics, factors significantly associated with all three CT use classes were mild to older age (30 + years), having 3 + comorbidities and history of having CT scan. Number of ED presentations and hospitalisations related to injury during follow-up period were also associated with all 3 CT scanning classes. However, the highest magnitude of association with all these factors was observed in class 1, “consistently high use”. (Table 4).

**Table 4:** Multinomial logistic regression models for the relationship between baseline demographic and clinical characteristics and membership of CT use classes in 3 years post injury

Characteristics	IRR	95%CI
Female		
No CT use	Ref	
Consistently high CT use Class 1	0.74	(0.54; 1.02)
Low CT use Class 2	1.08	(0.99; 1.17)
Temporally high CT use Class 3	0.81***	(0.73; 0.89)
30–44 years old		
No CT use	Ref	
Consistently high CT use Class 1	2.90*	(1.10; 7.61)
Low CT use Class 2	1.59***	(1.42; 1.78)
Temporally high CT use Class 3	1.33***	(1.18; 1.51)
45–64 years old		
No CT use	Ref	
Consistently high CT use Class 1	9.54***	(3.89; 23.3)
Low CT use Class 2	2.58***	(2.30; 2.90)
Temporally high CT use Class 3	1.90***	(1.67; 2.16)
65 + years old		
No CT use	Ref	
Consistently high CT use Class 1	18.8***	(7.53; 47.2)
Low CT use Class 2	4.33***	(3.76; 4.99)
Temporally high CT use Class 3	2.10***	(1.79; 2.47)
Less disadvantage SEIFA		
No CT use	Ref	
Consistently high CT use Class 1	1.03	(0.65; 1.63)
Low CT use Class 2	1.12	(1.00; 1.25)
Temporally high CT use Class 3	0.97	(0.85; 1.10)
Moderate disadvantage SEIFA		
No CT use	Ref	
Consistently high CT use Class 1	1.20	(0.81; 1.78)
Low CT use Class 2	1.21***	(1.09; 1.33)
Temporally high CT use Class 3	0.97	(0.86; 1.08)
High disadvantage SEIFA		
No CT use	Ref	
Consistently high CT use Class 1	1.00	(0.59; 1.70)
Low CT use Class 2	1.18*	(1.03; 1.36)
Temporally high CT use Class 3	0.99	(0.85; 1.15)
Highest disadvantage SEIFA		
No CT use	Ref	
Consistently high CT use Class 1	1.55	(0.80; 3.02)
Low CT use Class 2	1.28**	(1.08; 1.53)
Temporally high CT use Class 3	1.04	(0.86; 1.26)
Inner regional areas		
No CT use	Ref	
Consistently high CT use Class 1	0.90	(0.31; 2.61)
Low CT use Class 2	0.80	(0.59; 1.08)
Temporally high CT use Class 3	0.84	(0.63; 1.13)
Outer regional areas		
No CT use	Ref	
Consistently high CT use Class 1	0.67	(0.20; 2.26)
Low CT use Class 2	0.83	(0.62; 1.12)
Temporally high CT use Class 3	0.73*	(0.55; 0.97)
Remote areas		
No CT use	Ref	
Consistently high CT use Class 1	0.99	(0.32; 3.04)
Low CT use Class 2	0.84	(0.58; 1.20)
Temporally high CT use Class 3	0.91	(0.66; 1.24)
Very remote areas		
No CT use	Ref	
Consistently high CT use Class 1	1.00E-06	(0; 0.00)
Low CT use Class 2	0.63	(0.33; 1.17)
Temporally high CT use Class 3	0.99	(0.61; 1.60)
Head injury		
No CT use	Ref	
Consistently high CT use Class 1	3.19***	(2.16; 4.72)
Low CT use Class 2	1.00	(0.90; 1.12)
Temporally high CT use Class 3	3.91***	(3.50; 4.36)
Neck injury		
No CT use	Ref	
Consistently high CT use Class 1	1.46	(0.51; 4.18)
Low CT use Class 2	1.08	(0.85; 1.38)
Temporally high CT use Class 3	3.59***	(2.90; 4.44)
Thorax injury		
No CT use	Ref	
Consistently high CT use Class 1	1.82	(1.00; 3.34)
Low CT use Class 2	1.10	(0.92; 1.32)
Temporally high CT use Class 3	1.89***	(1.56; 2.30)
Abdomen injury		
No CT use	Ref	
Consistently high CT use Class 1	1.94*	(1.12; 3.34)
Low CT use Class 2	1.17	(0.98; 1.38)
Temporally high CT use Class 3	2.29***	(1.92; 2.72)
Multiple injury		
No CT use	Ref	
Consistently high CT use Class 1	2.77**	(1.30; 5.93)
Low CT use Class 2	1.08	(0.82; 1.42)
Temporally high CT use Class 3	2.16***	(1.70; 2.76)
Admitted to hospital		
No CT use	Ref	
Consistently high CT use Class 1	0.59*	(0.38; 0.93)
Low CT use Class 2	0.61***	(0.53; 0.71)
Temporally high CT use Class 3	2.39***	(2.05; 2.78)
External cause of injury: *Transport/pedestrian*		
No CT use	Ref	
Consistently high CT use Class 1	1.97*	(1.14; 3.41)
Low CT use Class 2	1.15	(0.98; 1.35)
Temporally high CT use Class 3	1.44***	(1.24; 1.67)
External cause of injury: *Force*		
No CT use	Ref	
Consistently high CT use Class 1	0.74	(0.43; 1.27)
Low CT use Class 2	0.97	(0.87; 1.09)
Temporally high CT use Class 3	0.50***	(0.43; 0.57)
Severity of injury: Moderate 94.1–99% SRR		
No CT use	Ref	
Consistently high CT use Class 1	1.57	(0.92; 2.67)
Low CT use Class 2	0.93	(0.73; 1.19)
Temporally high CT use Class 3	1.51***	(1.25; 1.81)
Severity of injury: Severe/very severe < = 95% SRR		
No CT use	Ref	
Consistently high CT use Class 1	0.48*	(0.26; 0.88)
Low CT use Class 2	0.49***	(0.37; 0.64)
Temporally high CT use Class 3	0.89	(0.72; 1.10)
Date of presentation: Weekend/PH		
No CT use	Ref	
Consistently high CT use Class 1	0.76	(0.54; 1.07)
Low CT use Class 2	0.95	(0.88; 1.03)
Temporally high CT use Class 3	1.05	(0.96; 1.15)
ED shifts: Evening 16:00–23:59		
No CT use	Ref	
Consistently high CT use Class 1	1.16	(0.84; 1.62)
Low CT use Class 2	0.94	(0.86; 1.02)
Temporally high CT use Class 3	1.04	(0.95; 1.15)
ED shifts: Night 0:00–7:59		
No CT use	Ref	
Consistently high CT use Class 1	0.61	(0.34; 1.08)
Low CT use Class 2	0.94	(0.83; 1.06)
Temporally high CT use Class 3	0.95	(0.83; 1.08)
Model of arrival: Ambulance		
No CT use	Ref	
Consistently high CT use Class 1	1.22	(0.84; 1.78)
Low CT use Class 2	1.01	(0.91; 1.13)
Temporally high CT use Class 3	1.44***	(1.29; 1.60)
Length of stay		
No CT use	Ref	
Consistently high CT use Class 1	1.03***	(1.02; 1.04)
Low CT use Class 2	0.99	(0.97; 1.00)
Temporally high CT use Class 3	1.03***	(1.02; 1.04)
History of comorbidity: 1–2 comorbidities		
No CT use	Ref	
Consistently high CT use Class 1	1.66	(0.95; 2.89)
Low CT use Class 2	1.43***	(1.30; 1.59)
Temporally high CT use Class 3	1.11	(0.99; 1.24)
History of comorbidity: 3 + comorbidities		
No CT use	Ref	
Consistently high CT use Class 1	3.05***	(1.87; 4.98)
Low CT use Class 2	1.79***	(1.60; 1.99)
Temporally high CT use Class 3	1.33***	(1.18; 1.51)
History of 1–2 hospitalisations		
No CT use	Ref	
Consistently high CT use Class 1	1.70*	(1.06; 2.73)
Low CT use Class 2	1.16**	(1.04; 1.30)
Temporally high CT use Class 3	1.34***	(1.17; 1.54)
History of 3 + hospitalisation		
No CT use	Ref	
Consistently high CT use Class 1	2.43**	(1.38; 4.27)
Low CT use Class 2	1.03	(0.85; 1.24)
Temporally high CT use Class 3	1.18	(0.96; 1.45)
History of CT scan		
No CT use	Ref	
Consistently high CT use Class 1	4.95***	(3.52; 6.97)
Low CT use Class 2	2.10***	(1.84; 2.39)
Temporally high CT use Class 3	1.86***	(1.60; 2.16)
Number of ED presentations related to injury		
No CT use	Ref	
Consistently high CT use Class 1	4.95***	(1.56; 1.98)
Low CT use Class 2	2.10***	(1.40; 1.55)
Temporally high CT use Class 3	1.22***	(1.15; 1.30)
Number of hospitalisations related to injury		
No CT use	Ref	
Consistently high CT use Class 1	1.78***	(1.61; 1.97)
Low CT use Class 2	1.51***	(1.43; 1.59)
Temporally high CT use Class 3	1.47***	(1.39; 1.55)

Whilst there was no factor associated with both class 1 and 2 (except for severe/very severe injury) or both class 2 and 3, several factors were associated with both class 1 and 3 including head injury, multiple injury, moderate level of injury and length of stay of the index hospital admission. However, the magnitude of the association was different. Multiple injury and moderate level of injury had the highest association with class 1, whilst head injury had the highest association with class 3 and length of stay was equally associated with both classes 1 and 3.

There was no factor uniquely associated with class 1. In contrast, neck injury, thorax injury, abdomen injury, traffic injuries and injury arrived by ambulance were uniquely associated with class 3. Living in an area of moderate to highest socio-economic disadvantage was only associated with class 2.

## Discussion

Our study found a third of patients with injury have at least one CT scan at any time over the 3-year follow-up. Using an advanced classification approach, instead of assuming a single pattern of CT use applied to all injury patients, this study has provided more nuanced understanding of the underlying patterns of CT use that may be useful for developing targeted interventions. Whilst we normally expect that CT scanning would be concentrated during an acute episode of injury (*i.e.* in ED and hospital), only half of the patients with an incident injury were found to follow that pattern. The remainder (of those who had a CT) had a substantial number of CT scans performed sometime after the index injury. This study found that nearly 10% of the total CT scans were performed on only 1% of the injured subjects. This group had an average of nearly 8 CT scans done within two and a half years. A recent meta-analysis found that cancer risks increased rapidly for radiation exposures above 55 mSv (which corresponds to the amount patients may get from 3 or more CT scans) [29]. For each additional CT scan, the risk of cancer increased by 0.16 (95% CI 0.13–0.19) [30]. Literature data also indicate that multiple CT scans within a short period of time may represent potentially avoidable CT scan procedures [31]. Therefore, identification of the consistently high-use group and factors associated with persisting high use in our study can contribute to raising awareness in clinical practice about the potential risk of excessive radiation exposure in this sub-population. Caution is needed when requesting additional CT scans for the high-risk group to minimise the risk of unnecessary radiation exposure.

Although CT scanning has become the screening test of choice for most injuries, its increasing use has been particularly concerning as injury patients tend to be relatively younger, hence, more radiosensitive [39]. On average, the cumulative effective dose is 2.6 milli-Sieverts (mSv) per injured patient per ED visit [40]. Studies found that the increasing use of CT scans in patients with injury does not correspond with improved patient outcomes [14, 15, 41]. However, findings from previous studies may have overlooked and underestimated the use of CT because of the restriction to a short emergent period (*i.e.* ED presentation) which failed to account for different patterns/trajectories of CT use over the course of injury management. Our study found only half of injured patients who had CT scanning had a peak of CT use occurring at the time of injury. This will enable the development of targeted interventions aimed at improvements in the efficiency of use of CT scanning for injured patients determining which patients should be the focus of radiation dose reduction strategies.

An interesting finding in our study is that moderate to highest disadvantage SEIFA were only significantly associated with low CT use class. Theoretically socio-economic factor should not affect access to CT scan whilst patients are in public hospitals which are fully funded by the State government. However, for CT scan performed out of hospitals, although they are subsidised under Medicare Benefits Scheme funded by the Federal government, there is an out-of-pocket payment for the cost above the Medicare reimbursement level. Since CT scanning is a relative expensive diagnostic imaging modality, the out-of-pocket cost can be a significant barrier for patients with lower socio-economic status. Patients with lower socio-economic status often live in more remote areas (i.e. less accessible areas) where lack of transport may also be a considerable barrier to access CT scanning. This is in line with our previous study which only examined the use of CT scanning in hospitals and suggested that accessibility (i.e. living in rural/remote areas) is a factor driving a reduction of CT scanning use. In this study, both CT scanning use in hospitals and out-of-hospital were captured, we provided further evidence that patients with lower socio-economic status rather than less accessibility (i.e. living in rural/remote areas) is a predictor of low CT use Western Australia after adjusting for other demographic and clinical characteristics. This finding is in line with many previous studies which indicated the association between low socio-economic status and low use of health care services [42, 43], such as specialist and the use of CT scanning [44].

The use of the advanced latent class mixture modelling applied in our study has advantages over the conventional cross-sectional approach to better capture high-need-high-cost-high-dose patients to improve efficient use and safety of healthcare resources and health outcomes. The latent class mixture modelling approach can further refine the prediction of individuals with a similar pattern, and in our study has successfully identified the top 1% of high CT use with an average of 8 CT scans per person over 3 years post injury. Using the cross-sectional approach obfuscates distinct patterns of CT use that reflect levels of intensity in exposure to medical radiation over time.

The high use of CT scanning in the consistent high use class may result in a small but considerable excess cancer risk. However, the risk of missing life-threatening injuries may outweigh the small long-term risk for cancer from imaging tests. A previous study suggested the median effective doses ranged from 2.1 mSv for a head CT to 31 mSv for a multiple abdomen and pelvis CT [45]. Another study in Western Australia examining 34 common CT scanning protocols found that the mean effective doses can be as low as 0.4 mSv for CT of the sinuses and as high as 31.2 mSv for CT of the whole body angiography [46]. About a third of CT protocols had mean effective doses greater than 10 mSv [46]. Our results are consistent with a recent systematic review investigating high-use-high-cost patients [47]. Our study provides further evidence to warrant intervention to avoid unnecessary CT scans, especially targeting at-risk subpopulations.

Several limitations should be considered when interpreting our findings. Whilst this study used linked whole-of-population health administrative data capturing the use of CT scanning both inside and outside hospital settings, CT scanning used in several secondary hospitals was not fully recorded. Due to incomplete records of CT use in secondary hospitals, this study was limited to injuries presenting to tertiary EDs in Western Australia. This limits generalising our findings to the whole population. Whilst the use of CT following the injury event was captured in all tertiary and most of secondary hospitals as well as private providers, the use of CT may be underestimated as the PACS data did not include all secondary hospitals. In addition, there is no information about the indication for the CT scan. Hence, it is possible that subsequent CT scans may not all be related to injury. This also prevented us from including the indication for the CT examination as a factor in predicting the CT use classes, although other proxy measures of clinical characteristics (i.e. comorbidity and history of hospital admission) were included, in addition to injury group and severity of injury. We cannot access the impact of the new injury on the use of CT scan. However, since the patients were followed up in a short time [3-year period], we assumed the chance of multiple new injuries occurred is minimal. We have included number of ED presentation and hospitalisations related to injury during the follow-up period to account for the potential effect. However, the rich source of longitudinal data has enabled us to fully explore the patterns of CT scanning use post injury and identify subpopulation with high use and risk due to the exposure to potentially large radiation doses. In addition, our study has broadly examined various factors associated with being classified in different classes of CT use that is informative for future interventions/policies targeting reduction of the unnecessary medical radiation exposure.

## Conclusion

Our broad exploration of the patterns of CT use 3-year post-incident injury will provide valuable information to assist with interpretation of findings in the current literature as well as to design future studies investigating the use of CT scans. It was found that the patients with the consistently high use of CT scan accounted for only 2.6% in the CT use groups but consumed substantial proportion of CT scans (10%). This study has also illustrated the use of a latent class mixture model in identifying distinctive and interpretable patterns of the CT use in patients with an incidence of injury. This method facilitates the identification of associated factors (within each class) which can be useful to design future policies/intervention targeting the high CT use sub-population to minimise the risk of exposure to medical radiation.

### Supplementary Information

Below is the link to the electronic supplementary material.

Supplementary file1 (DOCX 19 KB)

## Data Availability

The data that support the findings of this study are available from the relevant data custodians of the study datasets. Restrictions by the data custodians mean that the data are not publicly available or able to be provided by the authors. Researchers wishing to access the datasets used in this study should refer to the Western Australian Data Linkage Unit and the Australian Institute of Health and Welfare.
