# Supplementary material for: Patterns of computed tomography utilisation in injury management: latent classes approach using linked administrative data in Western Australia
Source: Eur J Trauma Emerg Surg. 2023 Jun 15;49(6):2413–27. doi: 10.1007/s00068-023-02303-y (PMC10728237; doi:10.1007/s00068-023-02303-y)
Supplement: Supplementary file 1 — Supplementary file1 (DOCX 19 KB) [file 68_2023_2303_MOESM1_ESM.docx]

**Appendix 1**

**Patterns of computed tomography utilisation in injury management: latent classes approach using linked administrative data in Western Australia**

**Author information**

Ninh T. Ha^1^, PhD, [thi.ha@curtin.edu.au](mailto:thi.ha@curtin.edu.au)

Mark Harris^2^, PhD, [Mark.Harris@curtin.edu.au](mailto:Mark.Harris@curtin.edu.au)

Max Bulsara ^3,4^, PhD, [max.bulsara@nd.edu.au](mailto:max.bulsara@nd.edu.au)

Jenny Doust^5^, BMBS, PhD, [j.doust@uq.edu.au](mailto:j.doust@uq.edu.au)

Sviatlana Kamarova^1,6,7^, PhD, [sviatlana.kamarova@curtin.edu.au](mailto:sviatlana.kamarova@curtin.edu.au)

Donald McRobbie^8^, PhD, [donald.mcrobbie@adelaide.edu.au](mailto:donald.mcrobbie@adelaide.edu.au)

Peter O’Leary ^1,9,10^, PhD, [peter.oleary@health.wa.gov.au](mailto:peter.oleary@health.wa.gov.au)

Paul M. Parizel^11,12^, MD, PhD, [paul.parizel@uwa.edu.au](mailto:paul.parizel@uwa.edu.au)

John Slavotinek^13^, MD, [John.Slavotinek@sa.gov.au](mailto:John.Slavotinek@sa.gov.au)

Cameron Wright^1,14,15,16^, MD, [cameron.wright@curtin.edu.au](mailto:cameron.wright@curtin.edu.au)

David Youens^1,4^, [david.youens@curtin.edu.au](mailto:david.youens@curtin.edu.au)

Rachael Moorin^1,4^ PhD, [R.Moorin@curtin.edu.au](mailto:R.Moorin@curtin.edu.au)

**Affiliations**

1. Health Economics and Data Analytics, Curtin School of Population Health, Faculty of Health Sciences, Curtin University, Western Australia;
2. School of Accounting, Economics and Finance, Faculty of Business and Law, Curtin University, Western Australia
3. Institute for Health Research, University of Notre Dame, Western Australia;
4. Centre for Health Services Research, School of Population and Global Health, The University of Western Australia;
5. Australian Women and Girls’ Health Research Centre, School of Public Health, University of Queensland;
6. School of Health Sciences, University of Sydney, New South Wales;
7. Nepean Blue Mountains Local Health District, New South Wales, Australia;
8. School of Physical Sciences, University of Adelaide
9. Obstetrics and Gynaecology Medical School, Faculty of Health and Medical Sciences, The University of Western Australia, Perth, Western Australia, Australia
10. PathWest Laboratory Medicine, QE2 Medical Centre, Nedlands, Western Australia, Australia
11. Medical School, University of Western Australia, Perth, Western Australia;
12. Department of Radiology, Royal Perth Hospital, Victoria Square, Perth WA 6000, Australia.
13. SA Medical Imaging, SA Health and College of Medicine and Public Health, Flinders University, Adelaide, South Australia
14. Fiona Stanley Hospital, 11 Robin Warren Dr, Murdoch, Western Australia, Australia
15. Division of Internal Medicine, Medical School, Faculty of Health and Medical Sciences, University of Western Australia
16. School of Medicine, College of Health and Medicine, University of Tasmania, Hobart, Tasmania

***Corresponding author:**

Ninh T. Ha

Health Economics & Data Analytics, Curtin School of Population Health, Faculty of Health Sciences, Curtin University

GPO Box U1987, Perth, Western Australia, 6845

Email: [thi.ha@curtin.edu.au](mailto:thi.ha@curtin.edu.au)

Phone: +61 08 9266 5134

**Appendix 1. Latent class mixture models among injury incidences over three year follow-up**

| **Model** | **No. of classes** | **AIC** | **BIC** | **loglik** | **conv** | **npm** | **SABIC** | **entropy** | **%class1** | **%class2** | **%class3** | **%class4** |
| --- | --- | --- | --- | --- | --- | --- | --- | --- | --- | --- | --- | --- |
| Model 1 - Quadratic | 1 | -101447.1 | -101412.8 | 50728.6 | 1 | 5 | -101428.7 | 1.0 | 100.0 |  |  |  |
| Model 2 -Cubic | 1 | -103460.1 | -103418.9 | 51736.1 | 1 | 6 | -103438.0 | 1.0 | 100.0 |  |  |  |
| Model 3 | 2 | -109929.3 | -109853.8 | 54975.7 | 1 | 11 | -109888.8 | 0.9 | 52.1 | 47.9 |  |  |
| **Model 4** | **3** | **-111091.6** | **-110981.8** | **55561.8** | **1** | **16** | **-111032.6** | **0.9** | **2.6** | **51.1** | **46.4** |  |
| Model 5 | 4 | -112380.3 | -112236.1 | 56211.2 | 2 | 21 | -112302.9 | 0.9 | 43.4 | 45.9 | 2.4 | 8.2 |

** The models were conducted among individuals with at least 1 CT over three-year follow-up period*

*Link: linear*

*AIC: Akaike Information Criterion*

*BIC: Baysesian Information Criterion*

*loglik: maximum log-likelihood*

*npm: number of parameters*

*entropy: discriminatory power- the accuracy of classification of individuals into different latent classes*

*conv: 1- convergence satisfied; 2- convergence not satisfied*
